# The capacity of exosomes derived from adipose-derived stem cells to enhance wound healing in diabetes

**DOI:** 10.3389/fphar.2023.1063458

**Published:** 2023-09-21

**Authors:** Feiyu Cai, Wenjiao Chen, Ruomei Zhao, Yi Liu

**Affiliations:** Department of Burns and Plastic Surgery, and Wound Repair Surgery, The Lanzhou University Second Hospital, Lanzhou, Gansu, China

**Keywords:** diabetes, exosome, stem cell, tissue regeneration, wound

## Abstract

The slow healing and nonhealing of diabetic wounds have long posed challenges for clinical practitioners. In the presence of elevated glucose levels, the body’s regulatory mechanisms undergo alterations that impede normal wound healing processes, including cell proliferation, cytokine release, and growth factor activity. Consequently, the advancement of stem cell technology has sparked growing interest in utilizing stem cells and their derivatives as potential therapeutic agents to enhance diabetic wound healing. This paper aims to provide an academic review of the therapeutic effects of adipose-derived stem cell-EXOs (ADSC-EXOs) in diabetic wound healing. As a cell-free therapy, exosomes (EXOs) possess a multitude of proteins and growth factors that have been shown to be advantageous in promoting wound healing and mitigating the potential risks associated with stem cell therapy. By examining the current knowledge on ADSC-EXOs, this review seeks to offer insights and guidance for the potential application of EXOs in the treatment of diabetic wounds.

## 1 Introduction

The skin serves as the primary defense mechanism of the body against external stimuli, safeguarding normal organ function by shielding them from potential harm. In the case of chronic wounds such as diabetic wounds and immunogenic wounds, accurate assessment, an effective treatment approach, and the involvement of a multidisciplinary clinical team are of utmost importance (Mustoe et al., 2006; Hu et al., 2015; Han and Ceilley, 2017). In accordance with incomplete statistical data, the global diabetic population is estimated to be approximately 463 million individuals, constituting 9.3% of the total global population (Zhang et al., 2017). Furthermore, evidence indicates that 6.3% of diabetic patients experience varying degrees of wound ulceration (Zhang et al., 2017; Cuadros et al., 2021). Clinicians have persistently endeavored to address the challenge of impaired healing in diabetic wounds. Nevertheless, conventional wound treatment approaches, encompassing skin grafting, flap transplantation, laser treatment, and biological scaffolding, may entail various risks, including donor site injury, scar tissue reduction, and pigmentation issues (Orgill and Ogawa, 2014; Goodarzi et al., 2018). In recent years, the advancement of stem cell technology has presented novel strategies for wound healing. Research has demonstrated that stem cells possess the ability to facilitate wound healing through the regulation of the inflammatory response, augmentation of angiogenesis, promotion of fibroblast proliferation, modulation of collagen, and the crucial involvement of exosomes (EXOs) (Salem and Thiemermann, 2010; De Miguel et al., 2012; Yuan et al., 2014a; Trounson and Mcdonald, 2015; Hu et al., 2018). These polyvesicles, released by stem cells via paracrine secretion, significantly contribute to the functionality and efficacy of stem cells in wound healing processes (Riau et al., 2019). Several studies have demonstrated that EXOs possess the potential to yield comparable outcomes to those of stem cell therapy, while also circumventing numerous risks commonly associated with cellular therapy, including the initiation of tumorigenesis and morphological abnormalities (Burger et al., 2015; Chang et al., 2019; Xu et al., 2020).

Numerous benefits of EXOs have been substantiated through clinical applications and fundamental experiments, encompassing their facile preservation, manageable dosages, potent biological impacts, and absence of immunological rejection hazards (Xunian and Kalluri, 2020; Lv et al., 2022). As the field of stem cell technology has advanced, there has been extensive research conducted on the effects and mechanisms of stem cell derived EXOs. This research holds potential for the application of cell-free EXOs therapy in diverse fields. A study has provided evidence that EXOs can inhibit and regulate the Wnt signaling pathway, thereby facilitating the restoration of osteogenic differentiation in inflammatory periodontal membrane stem cells. This, in turn, can expedite bone formation in alveolar bone defects (Lei et al., 2022). Moreover, EXOs have demonstrated efficacy in the treatment of cutaneous injuries, with findings indicating their ability to enhance skin nerve regeneration through the recruitment of fibroblasts and subsequent stimulation of nerve growth factor secretion (Zhu et al., 2022). Numerous investigations have further revealed the capacity of EXOs to modulate immune responses, facilitate tissue generation, exhibit anti-tumor properties, and perform various other functions, thereby presenting significant research prospects for addressing intricate ailments (Melzer et al., 2020; Zhao et al., 2021a; You et al., 2021; Liang et al., 2022).

Several characteristics of adipose-derived stem cells (ADSCs), including their easy availability, high proliferation potential, self-renewal ability, and secretion of trophic factors and extracellular vesicles (EVs), make them a promising alternative to other sources of mesenchymal stem cells (MSCs), such as bone marrow-derived MSCs (BMSCs) (Mazini et al., 2019; Shukla et al., 2020). Research has demonstrated that ADSCs and their derivatives can exert paracrine signaling, which plays a crucial role in tissue regeneration by facilitating chemoattraction, angiogenesis, and prosurvival functions (Bajek et al., 2016). The success of ADSCs and their derivatives in tissue engineering has led to their prominent consideration as potential candidates. Consequently, this study primarily focuses on reviewing the role and associated mechanisms of ADSC-EXOs in facilitating wound healing, while also assessing the potential of EXOs as a therapeutic approach for wound healing in diabetic patients.

## 2 Mechanisms of diabetic wound healing

In the context of wound healing, numerous factors are implicated, culminating in the preservation of the skin’s structural integrity. The process of wound healing encompasses four distinct phases: hemostasis, inflammation, proliferation, and remodeling (Monaco and Lawrence, 2003; Jeschke et al., 2015; Han and Ceilley, 2017). Following appropriate wound management, acute wounds typically exhibit prompt healing and a reduced incidence of complications. Nevertheless, it is frequently observed that diabetes mellitus induces alterations in physiological functions, thereby impeding the customary course of wound repair and leading to delayed or nonhealing wounds.

### 2.1 Cell proliferation and secretion

**FIGURE 1:**
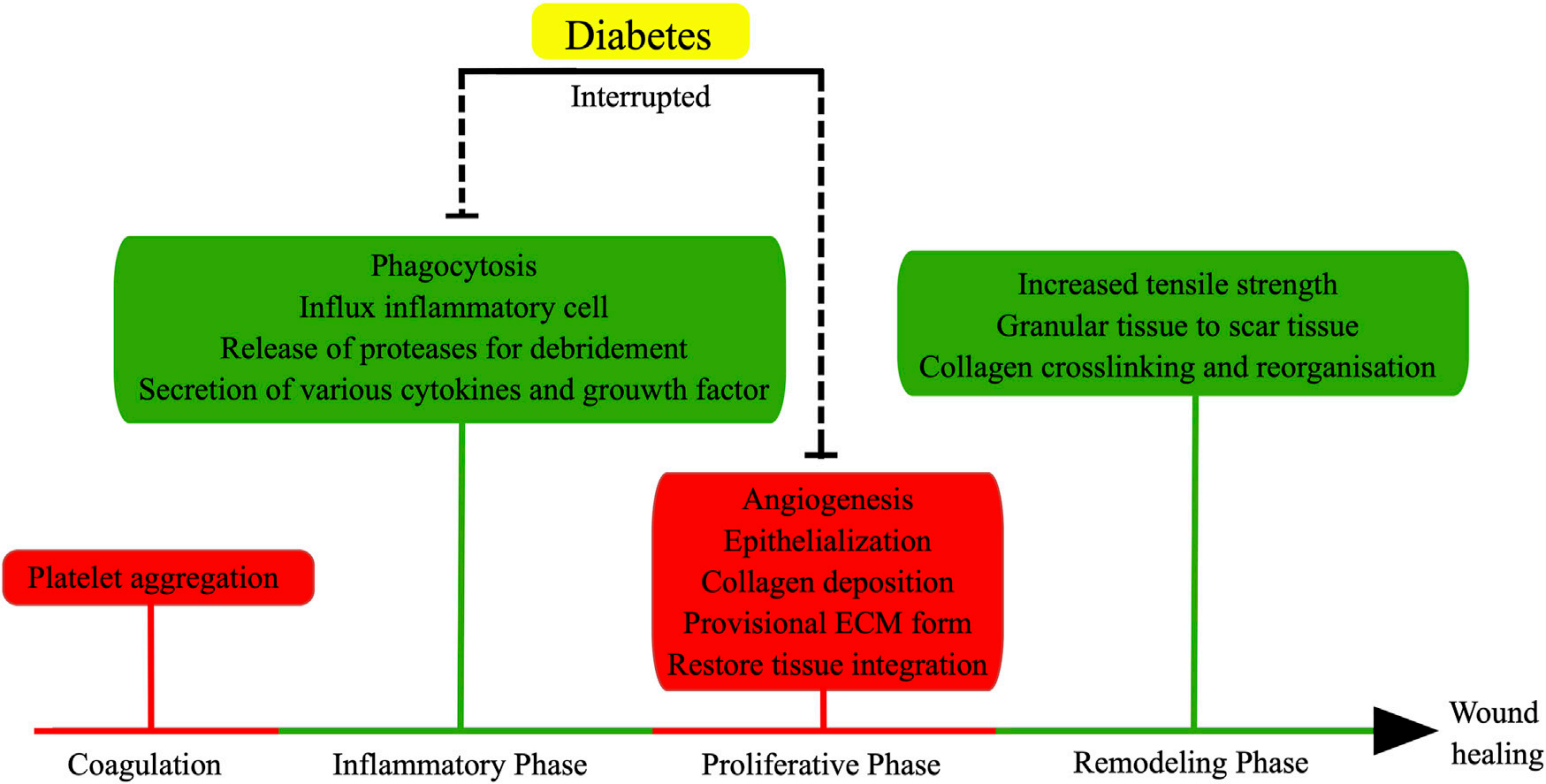
Normal wound healing involves four stages: coagulation, inflammatory phase, proliferative phase, and remodeling phase. Inflammatory and proliferative phases are the major factors to affect normal wound healing in diabetes. (the figure is drawn by Figdraw).

In the presence of elevated glucose levels, the healing process of typical wounds is adversely impacted, resulting in delayed or nonhealing wounds (Mustoe et al., 2006; Han and Ceilley, 2017) (Figure 1). Diabetic wounds, in particular, exhibit enduring inflammation, compromised tissue regeneration, and diminished resistance to mechanical forces, potentially attributable to vascular impairment and oxidative stress induced by ischemia/hypoxia (Alavi et al., 2014; Deng et al., 2021). Furthermore, heightened glucose environments impede the proliferation, differentiation, and secretion of cells crucial for wound healing, consequently prolonging the healing duration of wounds (Maruyama et al., 2007; Basu et al., 2018). For example, the transformation of monocytes into macrophages can contribute to the facilitation of wound healing through the secretion of diverse cytokines, including TNF-α, IL-6, IL-1β, and VEGF (Babaei et al., 2013). Nevertheless, the presence of hyperglycemia and oxidative stress can lead to macrophage dysfunction, resulting in a delay in the wound healing process (Maruyama et al., 2007; Basu et al., 2018). Furthermore, the investigation revealed that diabetic mice experienced delayed wound healing, accompanied by impaired migration and proliferation of keratinocytes and fibroblasts, as well as reduced levels of growth factors (Loot et al., 2002).

### 2.2 Immune regulation and heat shock proteins (HSPs)

Furthermore, the immune regulatory function, which is modulated by various inflammatory cells and secreted factors such as neutrophils, monocytes, T cells, B cells, and mast cells, also plays a significant role in the impaired healing of diabetic wounds. The accumulation of T cells within these wounds leads to elevated levels of TNF-α and other proinflammatory cytokines, ultimately disrupting the normal progression of the inflammatory cascade and contributing to excessive inflammation and insulin resistance (Moura et al., 2017). Moreover, mast cells are responsible for the secretion of angiogenic factors such as FGF, VEGF, and TGF-β1, which exert a crucial influence on vascular formation and regression during both the proliferative and remodeling phases of wound healing (Bevan et al., 2004; Nishikori et al., 2014; Tellechea et al., 2016). Additionally, the investigation revealed that in a murine model of inherited diabetes, the presence of mast cells and their secreted factors was diminished, resulting in a delay in vascular formation and regression (Bevan et al., 2004).

Heat shock proteins (HSPs) possess the ability to facilitate the process of wound healing through the recruitment of dermal fibroblasts, regulation of oxidative stress, and stimulation of cell proliferation in the presence of ischemic and hypoxic conditions (Luong et al., 2012; Singh et al., 2015). A study has demonstrated that diabetes mellitus hinders wound healing by diminishing the levels of HSP47, HSP70, and HSP90, which is achieved through the reduction of TLR4 and P38-MAPK expression (Park et al., 2018). Furthermore, there is evidence suggesting that HSP70 can serve as a biomarker and a potential target for intervention in diabetes mellitus, and it can also coexist in various cell types to regulate their respective activities (Krause et al., 2015). In the context of diabetes mellitus, the upregulation of HSP70 expression in the circulating blood (eHSP70) has been observed, while the extent of expression in local tissues (iHSP70) remains uncertain. The differential expression of eHSP70 and iHSP70 plays a significant role in the regulation of inflammation (Krause et al., 2015). It is evident that iHSP70 exerts inhibitory effects on inflammation, whereas eHSP70 promotes inflammation (Krause et al., 2015). Furthermore, HSP70 has been implicated in various diseases, including diabetes, as an immune-related response that serves as a danger signal under chronic stress conditions (Asea et al., 2002; Jheng et al., 2015). Based on the available evidence, numerous studies have been conducted to investigate the role of HSPs in EXOs. One study has demonstrated that tumor-EXOs exhibiting elevated levels of HSP90 can facilitate the migration and invasion of tumor cells (Zhang et al., 2020). Moreover, EXOs derived from plasma and expressing HSP70 have been found to enhance the survival of cardiomyocytes during ischemia-reperfusion injury by activating the ERK/p38MAPK/HSP27 signaling axis (Vicencio et al., 2015). Additionally, Ren et al. have provided further confirmation that adipose stem cell-derived microvesicles (ASC-MVs) promote wound healing by optimizing cellular function through the AKT and ERK signaling pathways, potentially involving HSP (Ren et al., 2019).

### 2.3 MicroRNA

MicroRNAs ranging from 19 to 24 nucleotides in length play a significant role in a range of physiological and pathological processes. Extensive research has elucidated the involvement of miRNAs in the repair of diabetic wounds and the promotion of wound healing through their regulatory functions (Li et al., 2021a; Li et al., 2021b; Dewberry et al., 2022; Hu et al., 2022). Specifically, during the healing process, miR-210 can be induced by hypoxia in diabetic wounds, leading to a decrease in keratinocyte proliferation (Biswas et al., 2010). Additionally, miR-210b can impede wound angiogenesis by modulating the expression of the globin transcription factor-2 (GATA-2) and the vascular endothelial growth factor receptor-2 (VEGFR-2) (Chan et al., 2012). Moreover, the genes miR-21, miR-26a, miR-130a, miR-146a, and miR-198 have been found to impede the process of wound healing in individuals with diabetes through the mechanisms of inflammation delay, cell proliferation inhibition, and diminished blood vessel formation (Bhattacharya et al., 2016; Icli et al., 2016).

### 2.4 Growth factors

**FIGURE 2:**
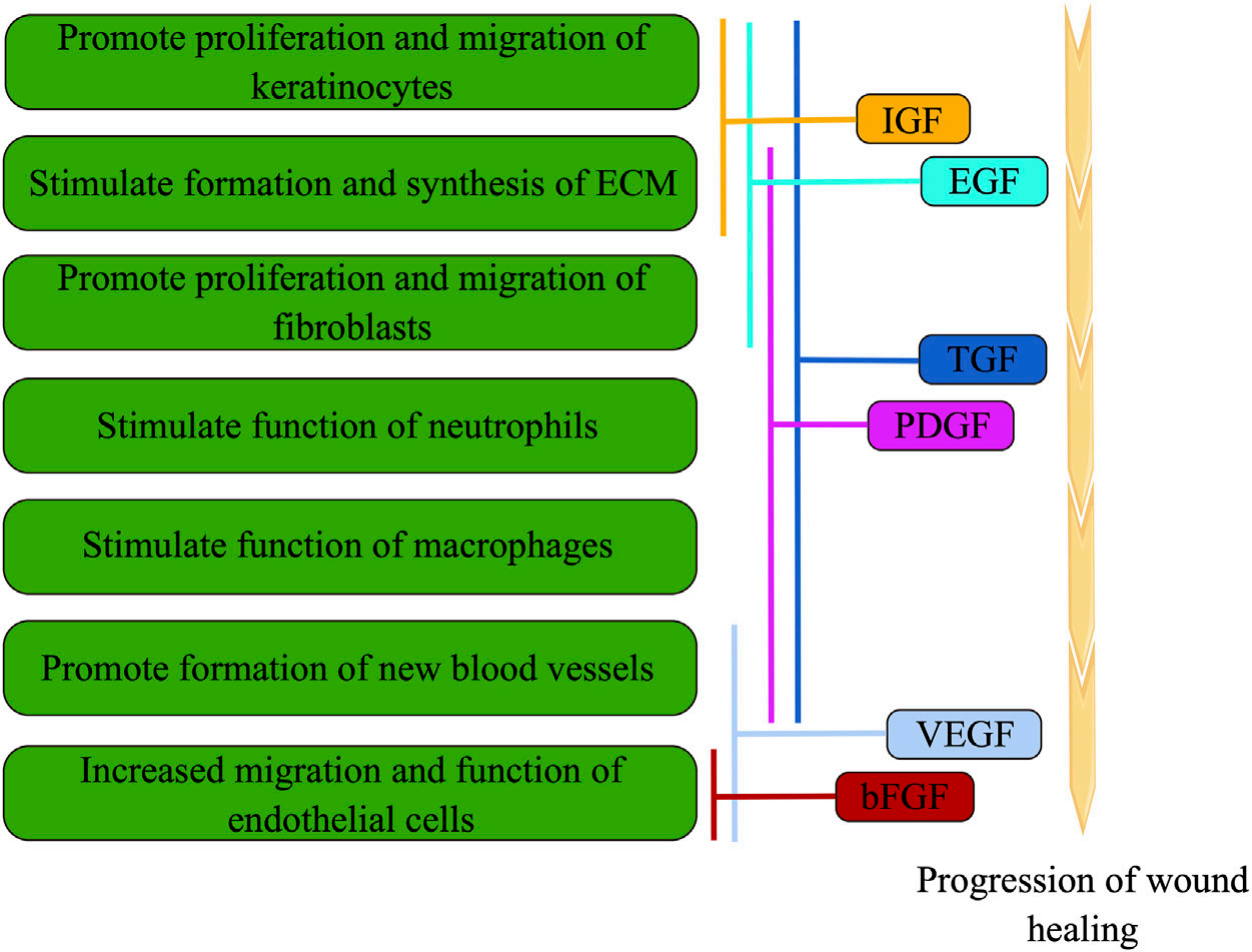
During wound healing, cell function is modulated by various growth factors through overlapping, cross-over, and synergistic interactions (the figure is drawn by Figdraw).

Furthermore, growth factors encompass bioactive proteins and peptides that play a crucial role in all phases of wound healing (Figure 2). However, in a hyperglycemic setting, the synthesis and breakdown of growth factors become disrupted, leading to a delay in the healing process. Specifically, platelet-derived growth factor (PDGF) is continuously synthesized and released by macrophages during wound repair. This, in turn, stimulates the migration and proliferation of fibroblasts, facilitating the production of proteins associated with granulation tissue, extracellular matrix (ECM), and blood vessels (Doxey et al., 1995). However, in the context of diabetic wounds, it is observed that the expression of PDGF decreases, indicating its involvement in the healing process of such wounds (Li et al., 2008). Moreover, the presence of basic fibroblast growth factor (bFGF) facilitates the proliferation and differentiation of fibroblasts, enhances the migration and functionality of vascular endothelial cells, thereby expediting the formation of granulation tissue and promoting wound healing (Tsuboi and Rifkin, 1990). Additionally, VEGF plays a crucial role in stimulating angiogenesis, a vital process for the regeneration and repair of diabetic wounds. According to a study, the upregulation of VEGF has been found to enhance blood flow and substance metabolism in diabetic wounds, facilitating adequate blood and oxygen supply for the healing process (Peng et al., 2021). Furthermore, the activation of VEGFR-1 has been associated with inflammation, whereas the activation of VEGFR-2 promotes angiogenesis (Angelo and Kurzrock, 2007). Previous research has indicated that diminished VEGF levels, elevated VEGFR-1 levels, and reduced VEGFR-2 levels may potentially impede the timely and effective healing of wounds (Zhou et al., 2015). In addition to the aforementioned growth factors, insulin-like growth factor (IGF), epidermal growth factor (EGF), and transforming growth factor (TGF) have demonstrated significant involvement in the process of wound healing (Figure 2).

## 3 The secretion and isolation of exosomes (EXOs)

### 3.1 The secretion of EXOs

**FIGURE 3:**
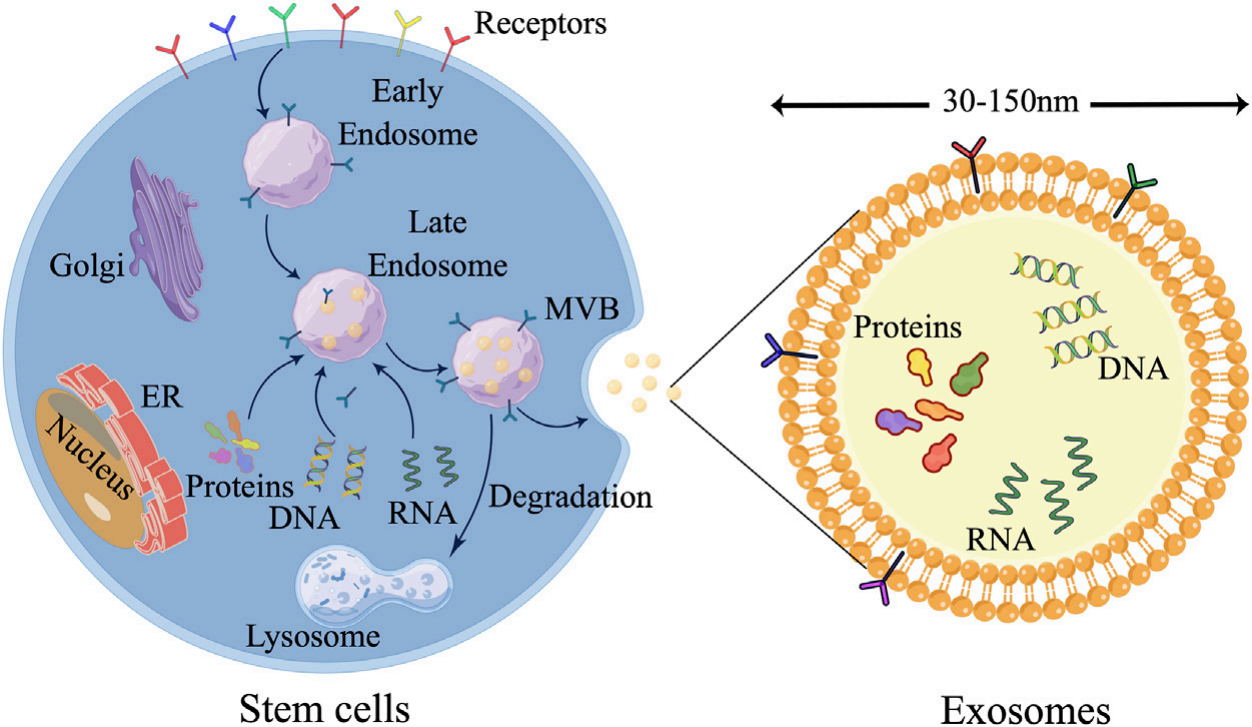
Three major steps are involved in the release of EXOs. 1) Primary endosomes are formed as the cell membrane invaginates, and early endosomes become late endosomes when the cell membrane acidifies. 2) Multivesicular bodies form as late endosomes bud inward. 3) Exocytosis releases exosomes into the extracellular environment after MVB and plasma membrane fusion. (the figure is drawn by Figdraw).

EXOs, which are characterized as cup-shaped cells with a diameter ranging from 30 to 150 nm, hold significant significance in cellular processes. These EXOs are produced through paracrine release with functional polyvesicles are released by cells into the body. The presence of EXOs can be detected in bodily fluids such as saliva, serum, and urine. EXOs encompass a diverse range of biomolecules including proteins, lipids, nucleic acids, and carbohydrates, and are actively involved in intercellular communication as well as extracellular matrix remodeling (Xunian and Kalluri, 2020; Lv et al., 2022). In the process of exosome release, three fundamental stages can be identified: (Lv et al., 2022) (Figure 3): Firstly, primary endosomes are generated as a result of the invagination of the cell membrane, and the transformation of early endosomes into late endosomes occurs upon acidification of the cell membrane. Secondly, multivesicular bodies are formed through the inward budding of late endosomes (Tiwari et al., 2021a). Lastly, exocytosis facilitates the discharge of EXOs into the extracellular milieu subsequent to the fusion of multivesicular bodies (MVB) with the plasma membrane (Gurunathan et al., 2021).

### 3.2 The technologies for isolating EXOs

Ultracentrifugation, size-based methodologies, and immunoaffinity capture represent a selection of conventional techniques employed for the isolation of EXOs. 1) Ultracentrifugation-based approaches exploit disparities in size and density among the constituents within the mixture solution to facilitate the extraction and isolation of EXOs (Saad et al., 2021). Two distinct forms of ultracentrifugation, namely, density gradient ultracentrifugation and differential centrifugation, are utilized for this purpose (Konoshenko et al., 2018; Gurunathan et al., 2019). Owing to its simplicity and cost-effectiveness, differential centrifugation is the more prevalent method employed for the separation of EXOs (Zhao et al., 2021b). 2) Size exclusion chromatography, a non-conventional separation technique, can be employed for the isolation of EXOs by adjusting the pore size to correspond with the exosome dimensions. During this procedure, the separation occurs through the diffusion of sample molecules smaller than the matrix pores into the matrix, while larger molecules are eluted (Tiwari et al., 2021b). Nevertheless, this approach encounters challenges in differentiating EXOs from microbubbles of identical size, resulting in suboptimal yields. 3) The immunoaffinity capture method entails the incorporation of diverse materials within antibodies, subsequently enabling the selective recognition of specific antigens present on the surfaces of EXOs (Li et al., 2021c; Kandimalla et al., 2021). Within this method, the identification of EXOs is achieved through the utilization of capturing antibodies, which exhibit specific recognition capabilities due to the embedded materials (Alzhraniet al., 2021). Although this approach enhances the purity of EXOs, it is accompanied by a significantly low yield (Fu et al., 2019).

## 4 ADSC-EXOs promote wound healing

**FIGURE 4:**
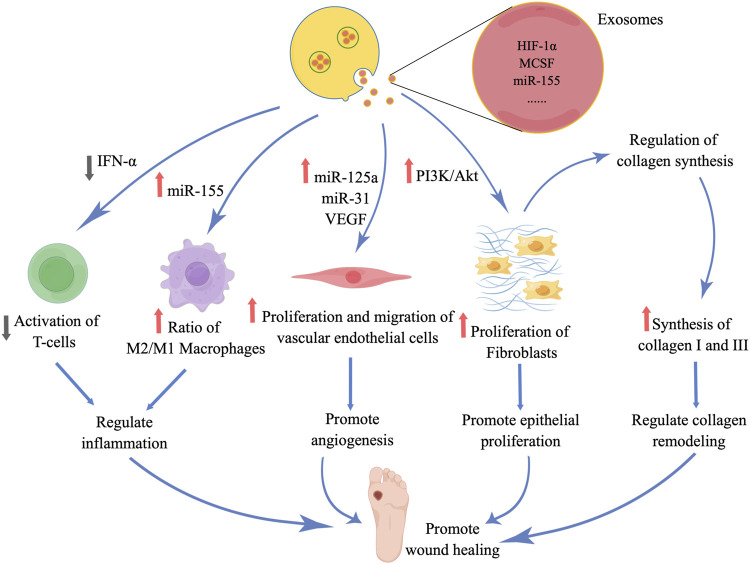
Diabetic wound healing is promoted by ADSC-EXOs, which regulate inflammation, stimulate angiogenesis, promote epithelial proliferation, repair, and remodel collagen. (the figure is drawn by Figdraw).

ADSCs possess several notable attributes, such as their ready accessibility, substantial proliferative capacity, ability to self-renew, and secretion of trophic factors and EVs. These characteristics have greatly facilitated the utilization of ADSC-EXOs in both scientific investigations and clinical applications (Mazini et al., 2019; Shukla et al., 2020). Numerous studies have substantiated the significance of ADSC-EXOs in the therapeutic management of diabetic wounds, as they regulate inflammation, promote angiogenesis, enhance epithelial proliferation and repair, and modulate collagen remodeling (Figure 4). Moreover, it is imperative to acknowledge that throughout different phases of wound healing, EXOs play a crucial role in modulating diverse cellular processes and facilitating the release of growth factors in a coordinated manner, thereby enhancing the wound healing process.

### 4.1 Regulate inflammation

The inflammation of a wound arises due to the release of growth factors and cytokines from blood vessels and platelets following skin trauma. Typically, inflammation can facilitate wound healing and possess anti-inflammatory characteristics. However, in cases where treatment is inadequate, local inflammation can hinder wound healing by eliciting an excessive inflammatory reaction and potentially leading to the dissemination of systemic infections (Yuan et al., 2014b). Consequently, healthcare professionals are progressively emphasizing the regulation of the inflammatory response in wounds to foster a conducive environment for healing and reconstruction. A study conducted revealed that ADSC-EXOs possess the ability to induce immunosuppressive effects, specifically inhibiting the proliferation of CD4^+^ and CD8^+^ T lymphocytes, through the reduction of interferon-α (IFN-α) secretion (Blazquez et al., 2014). This reduction in IFN-α secretion consequently leads to a decrease in inflammation, as observed in an *in vitro* experiment (Blazquez et al., 2014). Additionally, ADSC-EXOs were found to significantly diminish the production of IFN-γ in T lymphocytes, thereby suppressing the immune response (Blazquez et al., 2014). Given the crucial role of T lymphocytes and IFN-γ in immune-mediated inflammation, it can be inferred that ADSC-EXOs may serve as a vital factor in mitigating local hyperimmunity.

The polarization of macrophages is a critical factor in the process of wound healing, as it aids in the reduction of inflammation and the promotion of proliferation. However, the traumatic environment experienced by diabetic patients hinders the transformation of M1 macrophages into M2 macrophages, leading to prolonged inflammation and impaired wound healing (Bannon et al., 2013). Research has shown that ADSC-EXOs can facilitate the conversion of monocytes into M1 macrophages by upregulating miR-155 and mediating chronic inflammation (Zhang et al., 2016). Furthermore, EXOs have the ability to induce the transformation of macrophages into M2 phenotype, thereby facilitating wound healing through modulation of the M2/M1 ratio and enhancement of anti-inflammatory gene expression (Dalirfardouei et al., 2019). Additionally, EXOs exhibit the capacity to upregulate the expression of macrophage inflammatory protein-1α and monocyte-macrophage chemoattractive protein-1, thereby promoting the initiation of early inflammation during the wound healing process (Chen et al., 2019).

### 4.2 Promote angiogenesis

Angiogenesis in the wound site plays a crucial role in facilitating inflammatory responses, as it facilitates the transportation of metabolic waste, provision of nutrients to regenerating tissues, and contributes to the overall process of wound healing. A conducted study has revealed that the overexpression of miR-21 in ADSC-EXOs effectively promotes angiogenesis through the activation of AKT and ERK signaling pathways, as well as the upregulation of HIF-1α and SDF-1 expression (An et al., 2019). Additionally, the secretion of EXOs by stem cells widely contains VEGF, a significant regulator of angiogenesis, and its expression levels are observed to increase with miR-21 overexpression (An et al., 2019; Deng et al., 2020; Zha et al., 2021; Deng et al., 2022). Furthermore, the incorporation of miR-125a and miR-31 into human ADSC-EXOs facilitates their transfer to vascular endothelial cells, thereby inducing vascular regeneration (Liang et al., 2016). Additionally, EXOs enhance the migration and sprouting of vascular endothelial tip cells (Sharghi-Namini et al., 2014). In the context of diabetic patients, impaired local vascular regeneration is primarily responsible for inadequate wound healing. Cumulative evidence supports the notion that EXOs transport diverse proteins and growth factors, which in turn regulate angiogenesis and facilitate wound reconstruction. Furthermore, it has been observed in a study that the expression of VEGF was significantly increased in ADSC-EXOs when subjected to hypoxic conditions (Han et al., 2019). Consequently, investigating methods to enhance the therapeutic potential of EXOs in facilitating the healing of diabetic wounds through exogenous stimulation may prove to be a crucial avenue of research.

### 4.3 Promote epithelial proliferation and repair

The proliferation of fibroblasts plays a vital role in the late stage of wound healing, specifically in facilitating epithelial repair. During this stage, fibroblasts exhibit the ability to uptake and internalize ADSC-EXOs, leading to favorable outcomes such as enhanced migration, proliferation, and collagen synthesis (Hu et al., 2016). Additionally, the study demonstrates that the uptake of EXOs by fibroblasts is influenced by the dosage administered (Hu et al., 2016). Moreover, *in vivo* experiments conducted on mice involved pretreating ADSC-EXOs with endothelial differentiation medium (EDM), which resulted in increased secretion of EXOs and protein aggregation (Kang et al., 2016). It is widely acknowledged in the academic community that conditioned media contains cellular secretions, including growth factors and EVs. The findings of these studies provide further evidence supporting the significant role of EVs, including EXOs, in facilitating the transfer of growth factors between cells. The delivery of growth factors by EXOs, as depicted in Figure 2, has been observed to stimulate the proliferation, migration, and differentiation of various cell types. Consequently, the application of EDM-treated EXOs not only promotes angiogenesis but also enhances fibroblast characteristics and expedites the process of wound healing in the skin. Furthermore, *in vitro* studies have demonstrated that hypoxia-pretreated ADSC-EXOs possess the ability to modulate fibroblast proliferation and migration, as well as the synthesis of chemokines and extracellular matrix components in fibroblasts. These effects may be attributed to the activation of the PI3K/Akt pathway (Wang et al., 2021). Moreover, another investigation revealed that ADSC-EXOs exhibit a high abundance of micro-RNAs, with 199 of them being upregulated during exosome release. These micro-RNAs have been found to facilitate epithelial proliferation and facilitate tissue repair (Choi et al., 2018). Therefore, it is recommended to undertake additional research to enhance the upregulation of growth factors and miRNAs within EXOs via pretreatment intervention, aiming to modulate the cellular functionality involved in wound healing.

### 4.4 Regulate collagen remodeling

The determination of scar size and the ability of a repaired wound to withstand adequate tension are contingent upon collagen remodeling. In numerous instances, the hyperplastic nature of scars, which detrimentally impacts wound aesthetics and organ functionality, can be attributed to suboptimal collagen remodeling. However, the early administration of ADSC-EXOs has been observed to regulate collagen remodeling by fostering the synthesis of type I and III collagen, thereby facilitating a more robust process of wound healing (Hu et al., 2016). In a comparable manner, during the advanced phase of collagen remodeling, the EXOs also possess the ability to impede collagen synthesis and diminish the formation of scars, thereby contributing to the protection of organ functionality (Hu et al., 2016). Moreover, ADSC-EXOs have the ability to impede the transformation of fibroblasts into myofibroblasts, augment the proportion of transforming growth factor-β-3 to transforming growth factor-β-1 *in vivo*, and heighten the expression of matrix metalloproteinases-3 by means of activating the ERK/MAPK pathway. This activation leads to an increase in the ratio of matrix metalloproteinases-3 to tissue inhibitor of matrix metalloproteinases-1, thereby facilitating the restructuring of extracellular matrix and collagen, as well as promoting the progression of wound healing (Wang et al., 2017; Wang et al., 2021b). However, the mechanism by which ADSC-EXOs regulate collagen remodeling in diabetic wounds remains uncertain. Therefore, a comprehensive comprehension of the involvement of EXOs in collagen remodeling in diabetic wounds necessitates additional investigation.

## 5 Application method of EXOs

In recent years, several studies have employed topical subcutaneous injections of EXOs as a therapeutic intervention for diabetic wounds, yielding favourable outcomes. In particular, we conducted a comprehensive examination of applications for EXOs and evaluated their impact on the process of wound healing.

Various types of scaffolds, such as hydrogels, are frequently employed in tissue engineering investigations as vehicles for stem cells, growth factors, and pharmaceuticals. By encapsulating EXOs within hydrogels, their concentration at the site of injury can be efficiently enhanced, preventing dispersion and diffusion within bodily fluids, thereby extending their therapeutic duration (Wang et al., 2019). Moreover, hydrogels can also function as a filler to address soft tissue deficiencies in wounds and as a safeguard during the process of wound granulation (Wang et al., 2019). Chitosan, a widely employed biological material in hydrogel formulation, has found extensive application in tissue engineering endeavors. In a study conducted by Shi et al., chitosan/silk hydrogel sponges were utilized as carriers for EXOs in the treatment of diabetic skin defects. The findings of this investigation revealed that the combined utilization of hydrogel sponges and EXOs effectively facilitated collagen deposition and remodeling, augmented angiogenesis, and stimulated neuronal growth in diabetic rats, thereby fostering the process of wound healing (Shi et al., 2017). Another study demonstrated that the encapsulation of EXOs in nanohydrogel can induce an upregulation of VEGF expression via the ERK1/2 signaling pathway, thereby facilitating angiogenesis and promoting the healing process of diabetic wounds (Zhang et al., 2021). Moreover, the utilization of injectable, self-healing, antibacterial exosome polypeptide hydrogels exhibited superior efficacy compared to EXOs alone in treating diabetic wounds, resulting in satisfactory regeneration of skin appendages and reduced formation of scar tissue (Wang et al., 2021c). Moreover, a multitude of studies have demonstrated the efficacy of hydrogel scaffolds infused with EXOs for the treatment of diabetic wounds. These studies have specifically highlighted the effectiveness of various hydrogel types, such as Pluronic F127 hydrogel (Yang et al., 2020), carboxymethylcellulose hydrogel (Huang et al., 2021), and matrix metalloproteinase degradable polyethylene glycol smart hydrogel (Jiang et al., 2022).

An alternative method that can be considered is the utilization of ADSC-EXOs in wound dressings, with the aim of enhancing the healing process of diabetic wounds through localized osmosis. Shiekh PA et al. conducted a study wherein they assessed the efficacy of OxOBand, a wound dressing that incorporated ADSC-EXOs and possessed a high level of porosity, combined with antioxidant polyurethane, for the treatment of diabetic wounds. The findings demonstrated that compared to the control group, the utilization of OxOBand resulted in significant improvements in wound closure, collagen deposition, epithelialization, angiogenesis, and a reduction in oxidative stress (Shiekh et al., 2020). Moreover, the investigation additionally revealed that the implementation of OxOBand fosters the development of fully formed epithelial structures, resulting in regenerated skin with hair follicles and epidermis resembling those of healthy skin (Shiekh et al., 2020). Furthermore, an alternative approach involving intravenous injections of EXOs has been demonstrated to effectively mitigate scar hyperplasia in wounds (Wang et al., 2017). Nevertheless, further investigation is necessary to ascertain the precise means by which EXOs accurately target and function within the wound, as well as the potential underlying mechanisms involved.

## 6 Conclusion

In summary, the utilization of ADSC-EXOs holds promise for enhancing the healing process of diabetic wounds by modulating inflammatory responses, facilitating the formation of new blood vessels, stimulating the growth of epithelial cells, and regulating the restructuring of collagen. Furthermore, there exists significant potential for the development of therapeutic approaches that involve the direct application of EXOs as treatment or the manipulation of cellular secretion and responsiveness to these bioactive molecules in order to improve wound healing outcomes. However, several issues still require attention. Firstly, the animal models commonly employed to study diabetes do not accurately replicate the pathological progression observed in diabetic patients. Secondly, the large-scale production and clinical implementation of EXOs present ongoing challenges. Lastly, it is worth contemplating whether stem cells and their derivatives, such as EXOs and MVs, cultured in a simulated diabetic microenvironment *in vitro*, can enhance the healing process of diabetic wounds.
